# Predictors of placental malaria in Upper West Regional Hospital-Ghana

**DOI:** 10.1186/s12884-021-03861-y

**Published:** 2021-05-26

**Authors:** Pascal Kingsley Mwin, Afreh Kuffuor, Kaamel Nuhu, Rafiq Okine, Chrysantus Kubio, Frederick Wurapa, Francis Adjei Osei, Edwin Afari

**Affiliations:** 1 Field Epidemiology and Laboratory Training Program, School of Public Health, University of Ghana, Legon, USA; 2 Ghana Health Service, Upper West Regional Health Directorate, Karaga, Ghana; 3 State University of New York - Cortland, Cortland, USA; 4 World Health Organization (WHO), Country Office for Ghana, Accra, Ghana; 5 Ghana Health Service, Karaga District Health Directorate, Karaga, Ghana; 6 Public Health Unit, Komfo Anokye Teaching Hospital, Kumasi, Ghana; 7 KNUST School of Public Health, Kumasi, Ghana

**Keywords:** Placental malaria, Pregnant women, Gravidity, Ante-Natal Care attendance, Ghana

## Abstract

**Background:**

Placental malaria (PM) poses life-threatening complications to pregnant women as they are at increased risk of maternal and perinatal morbidity and mortality associated with malaria. This study examined the factors associated with placental malaria in the Upper West Regional Hospital (UWR).

**Methods:**

A cross-sectional hospital-based study was carried out among pregnant women delivering at Upper West Regional Hospital. A cross-sectional screening survey was conducted from January 2019 to April 2019. Three hundred eligible mothers were consecutively recruited. A record review of their maternal and child history was assessed using a checklist. Placental blood samples were taken for microscopy to determine placental malaria parasitemia. Logistic regression analysis was done to determine the factors associated with placental malaria at 95 % confidence level.

**Results:**

The proportion of mothers with placental malaria was 7 % (21/300), (95 % CI, 4.3–10.5 %). *Plasmodium falciparum* was the only species identified in those with PM. Majority of the women 66.7 % (14/21) with placental malaria had parasite density in the range 501 to 5,000 parasites/µL. Obstetric and health service factors that were significantly associated with placental malaria were gravidity and antenatal care (ANC) attendance. Primigravida (aOR = 3.48, 95 %CI = 1.01–12.01) and having less than 4 ANC attendance (aOR = 9.78, 95 %CI = 2.89–33.11) were found to be significantly associated with placental malaria.

**Conclusions:**

The proportion of women with PM was relatively low. Primigravid mothers reporting less than 4 ANC visits had the highest risk of placental malaria. Expectant mothers should be encouraged to attend at least 4 ANC visits prior to delivery.

## Background

Malaria in pregnancy is a serious public health issue globally as it poses life-threatening complications to both the mother and the fetus. Globally, over 50 million pregnant women are at risk of malaria in pregnancy annually [1]. The burden of Malaria in pregnancy is highest in sub-Saharan Africa with over 25 million cases recorded annually [2], reflecting the endemicity of Malaria in the region as many women in this sub-region get infected with malaria during pregnancy [3]. Those who become symptomatic seek early treatment, but majority of the asymptomatic patients are only seen when they develop complications [4] which may affect the mother and/or fetus. Maternal complications from malaria include anemia, increased risk of postpartum hemorrhage, puerperal sepsis and hypoglycemic episodes during pregnancy, while fetal complications from Malaria in pregnancy include Intra-Uterine Growth Restriction (IUGR), Low Birth Weight (LBW), congenital malaria, spontaneous abortions, still births, preterm delivery and prematurity [5].

Placental malaria in sub-Saharan Africa is usually from infection with *Plasmodium falciparum* species [6]. Following infection of maternal erythrocytes, the parasites are transported to the placenta where they adhere to the intervillous spaces of the mother’s site of the placenta resulting in complications to the fetus and the mother.

Among the many documented factors that contribute to a high risk of placental malaria with *P. falciparum*, nulliparity poses a greater risk compared with multiparity [7]. This is because infection in pregnancy leads to expression of *P*. *falciparum* erythrocyte membrane binding protein (PFEMP1) known as Variant2 chondroitin sulfate antigen (VAR2CSA) which is peculiar to pregnant women only. The expression of this antigen enables parasitized red blood cells (RBC) bind to oncofetal chondroitin sulfate receptor in the placenta, resulting in sequestration of the parasites in the placenta [8]. Immunity to VAR2CSA is not acquired until subsequent pregnancies, conferring a higher risk of placental malaria on primigravida compared with multigravida.

The prevalence of placental malaria varies across geographical settings in Africa, ranging from 8 % [2] to 65 % [4] in a hospital-based studies conducted in Tanzania and Nigeria respectively. In addition to endemicity, the proportion of women with placental malaria in Africa varies depending on the availability and adherence to various interventions put in place to control malaria among pregnant mothers. Availability and adherence to interventions such as use of Insecticide-Treated bed Nets (ITN) during pregnancy, intake of 3 or more doses of Intermittent Preventive Treatment with Sulfadoxine-Pyrimethamine (IPTp-SP) and appropriate, early treatment of malaria during the first trimester are interventions to reduce malaria in pregnancy and placental malaria.

Specifically in Ghana, malaria in pregnancy accounts for 14 % of Out Patients Department (OPD) attendance, 11 % of hospitalized admission and 9 % of maternal deaths [9], with prevalence ranging from 35.9 % in rural areas in the southern sector [10] to 52 % in the northern sector [11]. As part of measures to mitigate the burden of malaria in pregnancy in Ghana, the Ministry of Health adopted the 5 dose-IPTp-SP for malaria prevention in pregnancy in addition to other measures such as provision ITNs for use during pregnancy. These interventions notwithstanding, the prevalence of malaria in pregnancy and placental malaria in Ghana remain high.

We assessed the proportion of mothers with placental malaria and the associated factors among pregnant women reporting to the maternity ward of Upper West Regional Hospital in Ghana. While similar studies have been conducted elsewhere, area-specific studies such as the current one are key to informing appropriate area specific interventions based on the scope and peculiar associated factors of the problem of placental malaria in the defined area.

## Methods

### Study design and population

 The study was a hospital based cross-sectional study with a quantitative approach conducted between January 2019 to April 2019 who were recruited at the Upper West Regional Hospital.

This study involved pregnant women who presented at the labor unit of the Regional Hospital for delivery. All pregnant women who delivered at the Upper West (Wa) Regional Hospital during the study period and consented to the study were included. Pregnant women who were Glucose 6 phosphate dehydrogenase (G6PD) deficient, less than 28 weeks at the time of delivery and those requiring specialized care for conditions such renal failure and heart disease in pregnancy were excluded from the study due to higher vulnerabilities during delivery and associated limitations in obtaining informed consent.

### Study setting

 The study was conducted at the Maternity Unit labor ward of the Upper West Regional Hospital, a 196 bed-capacity hospital with 49 beds for obstetrics and gynecology that serves as the major referral center for the region and neighboring Burkina-Faso. The labor ward conducts a monthly average of 200 deliveries.

The Upper West region has a tropical climate with temperatures ranging from as low as 22.6^o^C and to as high as 40.0^o^C during the year. The total population of the region is 786,050 of which women in the fertile age (WIFA) forms 24 % [12].

### Sample size and recruitment

The sample size was estimated with a PM prevalence of 52 % [11] reported in urban Ghana, confidence interval of 95 % which corresponds to 1.96 standard values, and a margin of error of 6 and 10 % non-response rate, the sample size was estimated at 293. After receiving ethical clearance from the Institutional Review Board of the Ghana Health Service, a total of 300 parturient mothers took part in the study after providing individual consent following review of the study protocols and approved ethical sanctions by the research assistants. The eligible mothers were recruited consecutively from the beginning of January 2019 to April 2019. All mothers who reported for delivery at the regional hospital during the study period, met the criteria and consented to the study were recruited consecutively till the 300th participant.

### Data and sample collection

A structured interview guide was designed and used to interview mothers after delivery. The questions included socio-demographics (maternal age, marital status, ethnicity, religion, educational status and occupation), the use of insecticide treated bed nets and indoor residual spraying (IRS) exposure among others. A checklist (designed with Enketo software) was used to collect maternal data from the maternal and child health records booklet of respondents. The variables included obstetric variables (Number of pregnancies - Gravidity and ANC attendance), medical history (HIV status, hypertension status and history of diagnosed Malaria in current pregnancy) and health service factors (use of Insecticide Treated Bed-Nets - ITN during pregnancy, Intermittent Preventive Treatment for Malaria - IPT during pregnancy and Indoor Residual Spraying against malaria causing mosquitoes during pregnancy - IRS exposure). The maternal records data were cross checked by asking the mothers the same questions, and any discrepancies reconciled or participants dropped if discrepancies could not be reconciled. A total of 4 research assistants (Midwives) were trained to administer the screening survey.

### Sample collection

Eligible participants had their placental blood taken immediately after delivery of the placenta. The delivered placenta was placed in a wide kidney dish with the maternal side facing upwards. A sterile soaked gauze with savlon was used to clean the maternal site of the placenta. An incision of about 1.5 cm deep was made in the mother’s part of the placenta through the intervillous space with a scalpel blade and a 5 cc syringe used to collect 3mls of placental blood from the pool of blood into ethylenediaminetetraacetic acid (EDTA) bottle.

### Microscopy

Thick and thin stains were prepared using 3 % Giemsa stain following standard protocol. The slides were read by a biomedical scientist under light microscope and those that were positive for malaria parasites were recorded in terms of parasite density (counts). For quality control purposes, the slides were read by two biomedical scientists. Where there were discrepancies, a third reading by an independent biomedical scientist was taken as the result. The placental malaria parasite density was also estimated. The determined malaria parasite density was further categorized into low parasite density, moderate parasite density and high parasite density according to the WHO classification. Low parasite density was malaria parasite density ≤ 500 parasites/µl, moderate parasite density was from 501 parasites/µl to 5,000 parasites/µl and high parasite density was > 5,000 parasites/µl. [13]Those samples that were positive also had the malaria parasite species recorded. The laboratory results were then matched with the maternal ID on the screening survey for entry into the Enketo software.

### Statistical analysis

Data cleaning was done in Microsoft Excel and later imported into STATA version 15 software (StataCorp. 4905 Lakeway Drive Station, Texas 77,845, USA) for analysis. Descriptive statistics was performed for all variables and expressed as frequencies and proportions. The quantitative variables were expressed as medians and interquartile range. The proportion of mothers with placental malaria was estimated via proportion of those respondents who tested positive for placental malaria. Using simple logistic regression, we tested the association between Placental Malaria (PM) and IPTp-SP intake, maternal age, gravidity and ITN use which we reported as crude odds ratios. Multivariable logistics regression models were fitted using backward stepwise approach to assess the effect of multiple factors on placental malaria. Independent Factors which yielded statistical significance were included in the model to generate adjusted odds ratio (AOR) with significance considered at 95 % CI.

## Results

### Characteristics of respondents

A description of the respondents is given in Table 1. The age of the participants ranged between 18 years to 48 years and the median age was 26 years (IQR = 22,31). 46 % of the participants (138/300) were between 25 and 34 years. (Table 1)

Table 2 provides the obstetric history obtained from the respondents and maternal records. Women who were pregnant for the first time (primigravida) formed 29.0 % (87/300) of the respondents. About half of the women had received three or more doses of the SP as intermittent preventive treatment for malaria in pregnancy. There were few respondents who never received SP throughout the pregnancy, 7 % (21/300). (Table 2)

**Table 1:** Socio- demographic characteristics of study subjects

Characteristic	Frequency (%)	Placenta Malaria	
		**Yes**	**No**
**Maternal age**			
18–24	126 (42.0)	16(76.19)	110(39.42)
25–34	138 (46.0)	4(19.05)	134(48.03)
35–44	34 (11.3)	1(4.76)	33(11.83)
≥ 45	2 (0.7)	0(0.0)	2(0.72)
**Marital status**			
Single	5 (1.7)	2(9.52)	3(1.08)
Married	282 (94.0)	14(66.67)	268(96.06)
Divorced	4 (1.3)	0(0.00)	4(1.43)
Co-habiting	9 (3.0)	5(23.81)	4(1.43)
**Ethnicity**			
Dagaare/Wala/Sisala	272 (90.7)	19(90.48)	253(90.68)
Frafra/Kassena/Builsa	6 (2.0)	2(9.52)	4(1.43)
Dagomba/Gonja/Mamprussi	13 (4.3)	0(0.00)	13(4.66)
Akan	3 (1.0)	0(0.00)	3(1.08)
Other	6 (2.0)	0(0.00)	6(2.15)
**Religion**			
Christianity	107 (35.7)	7(33.33)	100(35.84)
Islam	192 (64.0)	13(61.90)	179(64.16)
Traditional	1 (0.3)	1(4.76)	0(0.00)
**Educational status**			
No education	51 (17)	2(9.52)	49(17.56)
Basic Education	181 (60.3)	13(61.91)	168(60.21)
Secondary	39 (13)	4(19.05)	35(12.54)
Tertiary	29 (9.6)	2(9.52)	27(9.68)
**Occupation**			
Student/unemployed	53 (17.7)	4(19.05)	49(17.56)
Farmer/housewife	91 (30.3)	8(38.10)	83(29.75)
Business/civil servants	24 (8.0)	1(4.76)	23(8.24)
Trader	105 (35.0)	4(19.05)	101(36.20)
Other	27 (9.0)	4(19.05)	23(8.24)

**Table 2:** Obstetric and health service characteristics of study participants

Characteristics	Frequency (%)	Placenta Malaria	
		**Yes (%)**	**No (%)**
Gravidity			
Primigravida	87 (29.0)	13(61.90)	74(26.52)
Multigravida	213 (71.0)	8(38.10)	205(73.48)
IPT-SP Use			
No SP dose	21 (7.0)	4(19.05)	17(6.09)
Single dose	38 (12.7)	5(23.81)	33(11.83)
Two doses	91 (30.3)	7(33.33)	84(30.11)
Three doses	94 (31.3)	1(4.56)	93(33.33)
Four doses	39 (13.0)	2(9.52)	37(13.26)
Five doses	17 (5.7)	2(9.52)	15(5.38)
IRS			
Not Exposed	62(20.67)	12(57.14)	50(17.92)
Exposed	238(79.33)	9(42.86)	229(82.08)
ITN use			
Yes	178 (59.3)	9(42.86)	169(60.57)
No	122 (40.7)	12(57.14)	110(39.43)
ANC attendance			
None Attendant	4 (1.3)	1(4.76)	3(1.08)
1 to 3 attendance	32 (10.7)	12(57.14)	20(7.17)
4 or more attendance	264 (88.0)	8(38.10)	256(91.75)
Malaria in Index Pregnancy			
Yes	120(40.0)	15(71.43)	105(37.63)
No	180(60.0)	6(28.57)	174(62.37)
HIV status			
Negative	279 (93.0)	19(90.48)	260(93.19)
Positive	14 (4.7)	1(4.76)	13(4.66)
Not done	7 (2.3)	1(4.76)	16(2.15)
Hypertension (*n* = 299)			
Hypertension	7 (2.3)	3(15.00)	4(1.43)
Normotension	292 (97.7)	17(85.00)	275(98.57)

### Proportion of women with placental malaria

**Fig. 1:**
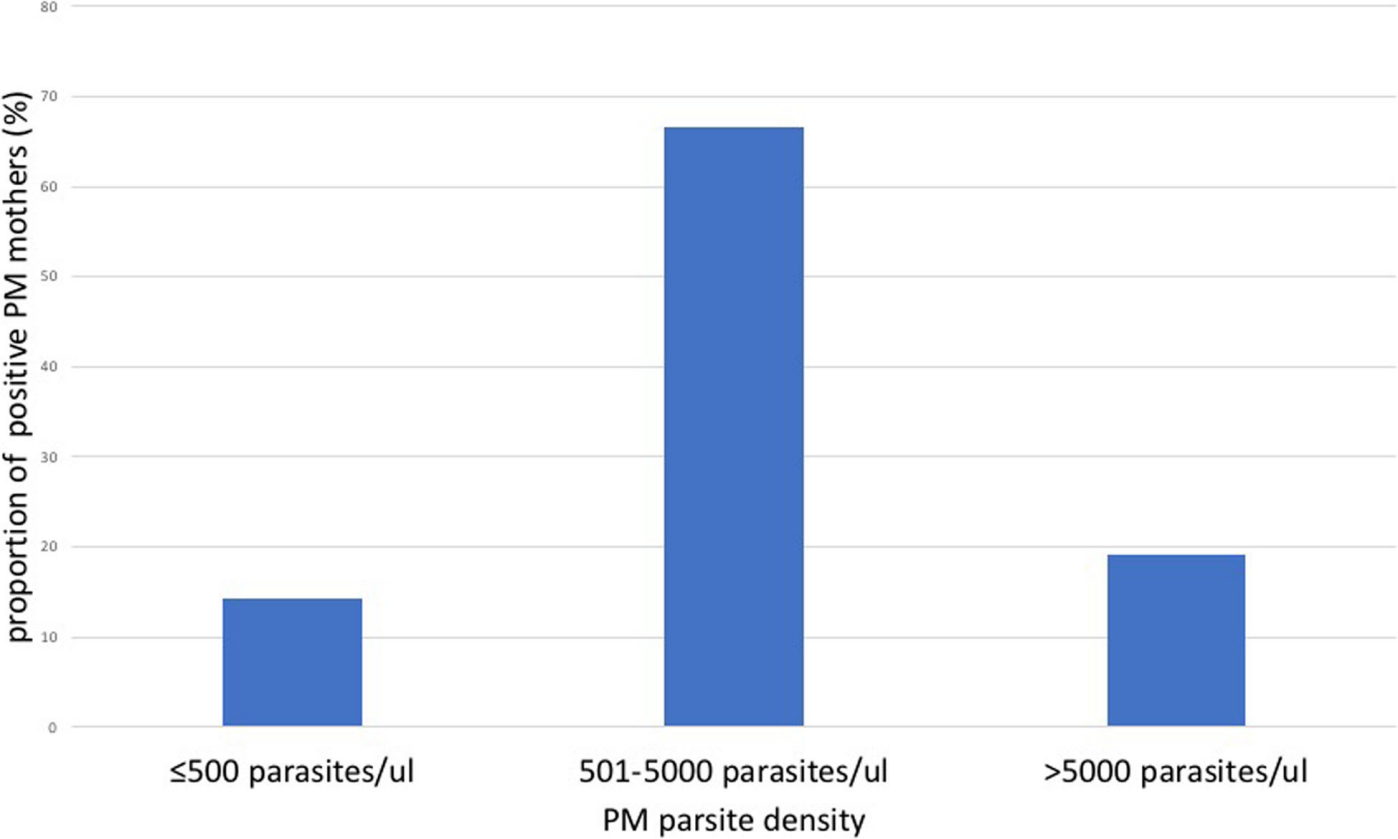
Placental Malaria (PM) parasite density of 21 mothers who tested positive

The proportion of placental malaria parasites among women delivering at the Upper West Regional Hospital was 7 % (21/300), (95 %CI, 4.3–10.5 %). All the placental malaria cases were *Plasmodium falciparum* species. The parasite density ranged from 237 parasites/ µl to 70,821 parasites/ µl. The median parasite density was 2,315 (IQR = 1101, 3860 parasites/µl). Figure 1 shows the parasite density of women whose placenta blood tested positive for malaria. (Fig. 1)

### Univariate factors associated with placental malaria

The association (bivariate analysis) between socio-demographic factors and placental malaria are presented in Table 3. The odds ratios indicate the likelihood of having placental malaria. The socio-demographic factors that were significantly associated with placental malaria were maternal age, marital status and ethnicity. Respondents who were 20 years or less had 6 times increase in odds of having placental malaria compared with respondents aged greater than 20 years (cOR = 6.04, 95 %CI = 2.42–14.09). Also, respondents who were not married had greater odds of placental malaria compared with those who were married (cOR = 12.18, 95 %CI = 4.09–36.21). (Table 3)

The association (bivariate analysis) between obstetric and health service factors and placental malaria are presented in Table 4. Respondents who took less than three doses of SP had greater odds of placental malaria compared with their counterparts who took three or more doses (cOR = 3.46, 95 %CI = 1.23–9.71). Primigravid mothers were four times more likely of having placental malaria compared with multigravida women (cOR = 4.5, 95 %CI = 1.79–11.29. Also, placental malaria was significantly associated with up to 3 ANC visits (cOR = 18.08, 95 %CI = 6.79–48.13), being hypertensive (cOR = 12.1, 95 %CI = 2.51–58.61), respondents with malaria in index pregnancy (cOR = 4.1, 95 %CI = 1.56–11.01) and respondents who were not exposed to IRS at their homes (cOR = 6.1, 95 %CI = 2.44–15.27). (Table 4)

**Table 3:** Analysis of socio-demographic factors associated with placental malaria

Characteristics	Placenta malaria		cOR (95 %CI)	*p*-value
	**No**	**Yes**		
**Maternal age**				
≤ 20 years	43(15.41)	11(52.38)	6.04(2.42–15.09)	< 0.0001*
* > 20 years*^a^	236(84.59)	10(47.62)	1	
**Marital status**				
Not Married	11(3.94)	7(33.33)	12.18(4.09–36.21)	< 0.0001*
* Married*^a^	268(96.06)	14(66.67)	1	
**Religion**				
Islam	179(64.16)	13(61.90)	1.04(0.40–2.69)	0.939
* Christian*^a^	100(35.84)	7(38.10)	1	
**Ethnicity**				
Frafra/Kasena/builsa	4(9.32)	2(9.52)	6.66(1.15–38.71)	0.035*
* Dagaare/wala/sisala*^a^	253(90.68)	19(90.48)	1	
**Educational status**				
None	49(17.56)	2(9.52)	0.55(0.07–4.14)	0.562
Basic	168(60.22)	13(61.91)	1.04(0.22–4.89)	0.956
Secondary	35(12.54)	4(19.05)	1.54(0.26–9.06)	0.631
* Tertiary*^a^	27(9.68)	2(9.52)	1	
**Occupation**				
Student/unemployed	49(17.56)	4(19.05)	0.47(0.11–2.05)	0.314
Farmer/housewife	83(29.75)	8(38.09)	0.55(0.15–2.05)	0.368
Business/civil servants	23(8.24)	1(4.76)	0.25(0.03–2.41)	0.231
Trader	101(36.21)	4(19.05)	0.23(0.05–0.98)	0.057
Other	23(8.24)	4(19.05)	1	

*-*P* < 0.05- *statistically significant*^a^*- reference groupcOR* Crude odds ratio

**Table 4:** Analysis of obstetric and health service factors associated with placental malaria

Characteristics	Placenta malaria		Crude OR (95 %CI)	*p*-value
	**No**	**Yes**		
**IPT-SP**				
< 3 doses	134(48.03)	16(76.19)	3.46(1.23–9.71)	0.018*
* ≥ 3 doses*^a^	145(51.97)	5(23.81)	1	
**Gravidity**				
Primigravida	74(26.52)	13(61.90)	4.5(1.79–11.29)	< 0.0001*
* Multigravida*^a^	205(73.48)	8(38.10)	1	
**ITN use**				
No	110(39.43)	12(57.14)	2.04(0.84–5.02)	0.117
* Usage*^a^	169(60.57)	9(42.86)	1	
**IRS**				
Not expose	50(17.92)	12(57.14)	6.1(2.44–15.27)	< 0.0001*
* Expose*^a^	229(82.08)	9(42.86)	1	
**HIV status**				
Negative	273(97.85)	20(95.24)	0.43(0.05–3.83)	0.457
* Positive*^a^	6(2.15)	1(4.76)	1	
**ANC visits**				
< 4 visits	23(8.24)	13(61.90)	18.08(6.79–48.13)	< 0.0001*
* ≥ 4 visits*^a^	256(91.76)	8(38.1)	1	
**Malaria in index pregnancy**				
No	174(62.37)	6(28.57)	1	
Yes	105(37.63)	15(71.43)	4.1(1.56–11.01)	0.004*
Hypertension				
Normotension	275(98.57)	17(80.95)	1	
Hypertension	4(1.43)	3(19.05)	12.1(2.51–58.61)	0.002*

*-*P* < 0.05- *statistically significant*^a^*- reference group* cOR Crude odds ratio

### Multivariable analysis of factors associated with placental malaria

The predictors (multivariable analysis) of placental malaria are presented in Table 5. ANC attendance of less than four visits was the strongest predictor for placental malaria (aOR = 9.78, 95 %CI = 2.89–33.11). Also, Primigravid mothers had increased likelihood of placental malaria (aOR = 3.48, 95 %CI = 1.01–12.01). (Table 5)

**Table 5:** Multivariable analysis of factors associated with placental malaria

Characteristics	aOR	95 %CI	p-value
**Gravidity**			
Primigravida	3.48	1.01–12.01	0.049*
* Multigravida*^1^			
**ANC visits**			
< 4 attendance	9.78	2.89–33.11	< 0.0001*
* ≥ 4 attendance*^1^			
**IPT-SP**			
< 3 doses	1.22	0.35–4.29	0.758
* ≥ 3 doses*^1^			
**Ethnicity**			
Frafra/Kasena/builsa	2.85	0.18–45.71	0.460
* Dagaare/wala/sisala*^1^			
**Marital status**			
Not Married	2.28	0.42–12.22	0.337
* Married*^1^			
**Maternal age**			
≤ 20 years	1.01	0.22–4.59	0.988
* > 20 years*^1^			
**Malaria in index pregnancy**			
Yes	1.64	0.47–5.69	0.436
No			
**Hypertension status**			
Hypertension	2.85	0.30–26.79	0.360
Normotension			
**IRS**			
No	1.86	0.49–7.11	0.362
Yes			

## Discussion

The study revealed a low proportion of mothers with placental malaria delivering at the Upper West Regional Hospital. In contrast, a high percentage (52 %) was recorded in a prospective study conducted by van Spronsen et al., [11] in the northern part of Ghana. The two study areas are similar in geographic location, ethnic background and climate. However, the difference in the prevalence could be due to malaria in pregnancy prevention interventions and differences in time between the two studies. It is however not surprising as Hommerich et al., [14] noted a 57 % decline in placental malaria in the southern part of Ghana between the years 2000 and 2006. The low proportion of women with placental malaria in this study could be a reflection of significant differences in gravidity, IRS exposure and 4 or more ANC visits as reported in Table 4. Although SP resistance is of increasing concern, it is still effective in preventing malaria during pregnancy as reported by several studies [15–17].

In this study, majority of the women (67 %) with placental malaria had parasite density in the range of 501 to 5000 parasites/µl. This is similar to a study conducted by Babalola et al., (2015) where microscopy technique was used to detect placental malaria with about 60 % of the positive samples having parasite density in the range of 501 to 5000 parasite/µl [18].

This study has shown that primigravida has a threefold increase in odds of placental malaria compared with multigravid women. This finding is consistent with several studies detailing the significant association between women who become pregnant for the first time and placental malaria compared with their counterparts entering their second or more pregnancy. A prospective cohort study on women delivering at a hospital in Papua New Guinea documented a two times increased in odds of placenta malaria in primigravid mothers compared with multigravida counterparts [6]. Ndeserua, Juma, Mosha, & Chilongola, (2015) also showed a significant higher risk of placental malaria among women in Tanzania in their cross-sectional study. In Nigeria, a study conducted by Babalola et al., [18] also agreed with this study as results of their study showed primigravid women having a significant increase in odds by two times compared with multigravid mothers.

It is a consensus from several researches that primigravid or nulliparous women are more susceptible to placental malaria. This stems from the fact that the immune system of primigravid women is being primed for the first time in the pregnancy in relation to *Plasmodium* parasite exposure. Placental malaria occurs when the *Plasmodium* infested red blood cell bind to chondroitin sulfate A (CSA) in the intervillous regions of the placenta leading to sequestration [19]. In secundigravida and multigravida, previous exposure to *Plasmodium* infection resulted in the production of antibodies that will bind to chondroitin sulfate A (CSA) in subsequent pregnancies thereby interfering with adhesion of infected *Plasmodium* red blood cells to CSA [20]. This phenomenon reduces the risk of placental malaria in multigravid women. Knowledge on primigravida being strongly associated with placenta malaria will help health educators emphasize the need for mothers being pregnant for the first time to regularly and timely attend antenatal services as well as adhere to malaria prevention and control strategies.

From this study, statistically significant association was found between women attending less than four antenatal visits and placental malaria. These women had 9 times increased odds of having placental malaria compared with women who met the WHO recommended visits. A study conducted in Sudan also reported 11 times increased odds of placental malaria among women who did not meet the recommended WHO ANC visits [21, 22]. This similar finding suggests that ANC attendance is very important in efforts aimed at controlling malaria in pregnancy. There is therefore the need for advocacy targeting pregnant women with the aim of achieving at least four ANC visits before delivery. Registering for ANC early in the pregnancy may be helpful in scheduling appropriate interval ANC visits towards realizing at least 4 ANC visits per pregnancy. To the extent that having 4 or more ANC visits was found to be associated with a lower burden of placental malaria in this study, it is conceivable that more ANC visits may have offered more opportunities for malaria intervention and control among participants with 4 or more ANC visits.

### Study limitations

Superior diagnostic method such as histopathology of placental tissue could improve the diagnostic capabilities and possibly increase the number of positive cases of placental malaria, hence giving a better picture of the prevalence.

We also recognize that while the overall study sample size of 300 participants was based on appropriate sample size estimations, the small yield of 21 PM cases (7 % of study participants), which was not within our control, may nevertheless limit the rigorousness of our inferential statistical analysis.

Additionally, while an alternative study design such as Case-Control study may have been better suited for this study, the lack of sufficient appropriate controls in the study area during the study period did not allow for such a design to be utilized.

## Conclusions

The study revealed a relatively low proportion of mothers with placental malaria among mothers who delivered at the Upper West Regional Hospital. Low ANC attendance as well as primigravida were strong predictors of placental malaria. This suggests improved ANC attendance may be beneficial in preventing placental malaria.

## Data Availability

The data collected for the study which has been analyzed and presented are available at the first authors’ institution and is available upon formal request.

## Abbreviations

PM: Placental malaria
IUGR: Intrauterine growth restriction
LBW: Low birth weight
PFEMP1P: Falciparum erythrocyte membrane binding protein
VAR2CSA: Variant2 chondroitin sulfate antigen
IPTp-SP: Intermittent Preventive Treatment in Pregnancy
OPD: Out Patients Department
ANC: Ante natal care clinic
ITN: Insecticide Treated Net
EDTA: ethylenediaminetetraacetic acid
WHO: World Health Organisation
GHS: Ghana Health Service
ERC: Ethics Review Committee
HIV: Human Immunodeficiency virus
GFELTP: Ghana Field Epidemiology and Laboratory Training Programme
CI: Confidence Interval
cOR: Crude Odds Ratio
aOR: Adjusted Odds Ratio
